# Theoretical Study of a Transition Metal-Modified B_12_N_12_ Nanocage for COCl_2_ Detection: Advances toward High-Sensitivity Materials for Phosgene Sensing

**DOI:** 10.1021/acs.langmuir.4c04850

**Published:** 2025-03-05

**Authors:** Natanael de Sousa Sousa, Jaldyr de Jesus Gomes Varela Junior

**Affiliations:** † Universidade Federal do Maranhão, 65080-805 São Luís, MA, Brazil

## Abstract

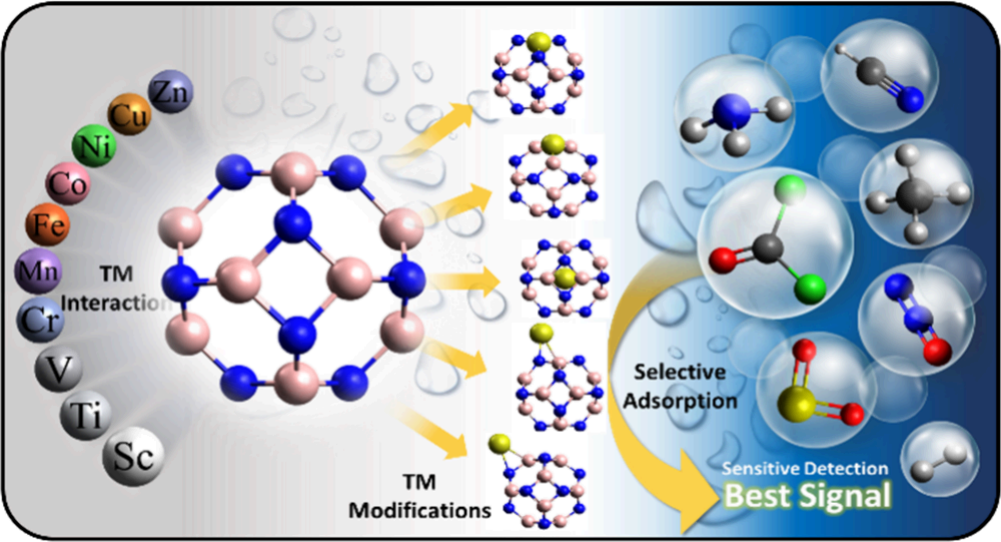

Phosgene gas (COCl_2_) is highly toxic and poses severe risks to human health and the environment. Its release can contaminate soil and water, disrupt ecosystems, and contribute to air pollution. This study employs density functional theory and time-dependent density functional theory calculations to explore the potential of pure and B_12_N_12_ nanocages modified with transition metals for phosgene detection. First-row transition metals (TM = Sc–Zn) were incorporated into the nanocages via five configurations: doped (TMB_11_N_12_ and B_12_N_11_TM), decorated (TM@b_64_ and TM@b_66_), and encapsulated (TM@B_12_N_12_). Geometric, electronic, and optical properties, charges, and adsorption energies were analyzed to understand the gas sensing properties. The results showed that phosgene weakly adsorbs on isolated B_12_N_12_ but preferentially binds via oxygen to the TM or boron atoms of the modified nanocages, undergoing dissociation in some interactions, such as in B_12_N_11_Sc and B_12_N_11_Ti, suggesting distinct adsorption mechanisms. TM modifications reduced the HOMO–LUMO gap, enhancing the conductivity and reactivity. Quantum descriptors identified Mn@b_64_ (TM decorated on a bond between four- and six-membered rings) as the most stable in the series, with Mn@b_64_ standing out for its high electronic sensitivity to phosgene, moderate adsorption energy (*E*_ads_ = −0.48 eV), and short recovery time (1.29 μs), which can be improved with an increase in temperature. The doped configuration B_12_N_11_Mn exhibited a stronger work function response (ΔΦ = 65%) than Mn@b_64_ (25%). Mn@b_64_ also demonstrated optical activity for COCl_2_ detection in UV–vis spectra and high selectivity against gases like H_2_, CH_4_, CO_2_, NH_3_, and H_2_S and water. Molecular dynamics (MD) confirmed the stability of the Mn@b_64_ system before and after phosgene adsorption. Compared with other systems in the literature, Mn@b_64_ exhibits better sensitivity and selectivity, even under high humidity or extreme temperatures. These results highlight its potential for developing high-performance, selective, and cyclic phosgene sensors.

## Introduction

Phosgene (COCl_2_) is a chemical compound with high toxicity and great historical and contemporary relevance. Discovered in 1812 by John Davy,^1^ phosgene gained notoriety during the First World War, when it was widely used as a chemical warfare agent due to its ability to cause serious or fatal injuries when attacking the respiratory tract.^2,3^ Phosgene is estimated to have caused about 80% of chemical deaths during the war.^3^ Its gaseous, colorless form and the characteristic odor of moldy hay are detectable only in dangerously high concentrations and aggravate the risks associated with its accidental or deliberate exposure.^4^

COCl_2_ is released during the combustion of carbon tetrachlorides, trichloroethylene, and other halogenated hydrocarbons.^5^ With a density of 4.24 kg/m^3^ at 25 °C and a boiling point of 8.3 °C, phosgene is a liquid at room temperature but volatile enough to vaporize quickly under normal conditions. Its inhalation causes the formation of hydrochloric acid in the lungs, resulting in edema and necrosis of lung tissue. Its toxicity derives from the ability to react with nucleophilic groups in biomolecules, such as amino acids and proteins, resulting in cellular and systemic damage and perhaps death due to respiratory failure.^6,7^ Furthermore, its persistence in the environment can cause damage to the flora and fauna, triggering significant ecological impacts. In soil and water, COCl_2_ reacts quickly with humidity, forming substances that contribute to the acidification of the environment, such as hydrochloric acid and CO_2_,^8^ which can damage aquatic and terrestrial ecosystems. Their safe handling and continuous environmental monitoring are therefore essential to prevent ecological catastrophes and protect public health.^9^

In recent decades, early and accurate detection of phosgene has become an attractive and necessary field of research. Innovative sensors, which combine high sensitivity and selectivity, have been developed to identify this gas in industrial and urban environments.^10−12^ For example, recent studies highlight the use of materials such as nanostructured metal oxides, conductive polymers, nanotubes, and two-dimensional (2D) materials to create high-efficiency sensors for polluting gases.^13,14^

A significant advance was reported by Xia et al.^15^ using a highly selective fluorescent probe constructed by linking *o*-phenylenediamine to coumarin (*o-Pac*), which responds to phosgene in turn-on fluorescence mode. The response time was found to be less than 0.5 min, and the detection limit was 3 nM. The sensor identified the COCl_2_ front triphosgene and various acyl chlorides and can be manufactured on paper with a polystyrene membrane containing *o-Pac* for selective real-time monitoring. In a new approach, Ravi and collaborators^16^ make use of surfactant-free 1,8-diaminonaphthalene (DAN)-functionalized graphene quantum dots (DAN-GQDs) to detect phosgene in an aqueous solution. Interaction with the phosgene molecule (0 → 88 μL) reduces the initial fluorescence intensity of DAN-GQDs (Φ*F*, 53.6% → 34.6%) through the formation of a stable six-membered cyclized product. The authors state that DAN-GQDs showed excellent selectivity and sensitivity and a detection limit of 2.26 ppb compared with other toxic pollutants in water.

In the theoretical field, Behestian et al.^17^ investigated the application of pure and Sc-doped boron nitride nanotubes (BNNTs) as a material for phosgene detection, using density functional theory (DFT) calculations. They verified that unlike isolated BNNTs, Sc-doped nanotubes can effectively interact with the phosgene molecule, such that their electronic properties and work functions (Φ) are drastically changed upon exposure to the gas. Idrees and collaborators^18^ analyzed the application of fullerene-like cages as a sensor for detecting phosgene and diphosgene. The detection capacity of the nanocages was evaluated by measuring the energy gap value, sensitivity, and recovery time of the complexes. The authors indicated that B_38_ has the highest sensitivity and the shortest recovery time. Therefore, they concluded that among the structures studied B_38_ is a more effective sensor for detecting the gas.

In a recent DFT approach, Deji and co-authors^19^ reported that ordered atomic doping led to an increased sensitivity of phosgene gas detection in graphene nanoribbons. They promoted the interaction of the COCl_2_ molecule with various B- and P-doped configurations at the edge and center of flat (AGNR) and zigzag (ZGNR) graphene nanoribbons. Their results showed that substitutional doping with P or B atoms in different positions had a significant impact on the structural, electronic, and adsorption properties of the nanoribbons. They observed that the highest adsorption energy (*E*_ads_ = −8.83 eV) was obtained for P-doped configurations.

Derived from fullerene (C_60_), the structure of B_12_N_12_ was initially proposed theoretically by Jensen and Toftlundt,^20^ consisting of eight hexagonal rings and six tetragonal rings, which is the most stable BN nanocluster, and its experimental synthesis, isolation, and modification were first developed by Oku et al. in 2004.^21^ We currently know that it is possible to obtain B_12_N_12_ from boron and nitrogen precursors,^22^ with plasma electrical discharge and plasma-assisted chemical vapor deposition. B_12_N_12_ nanocages have characteristics that include stability at high temperatures, a low dielectric constant, a high thermal conductivity, and resistance to oxidation, making it an excellent candidate for application in sensing.

Silva et al.^23^ investigated interactions of CNCl with MB_11_N_12_ (M = Fe, Co, Ni, Cu, or Zn) nanocages, reporting the most significant reduction in *E*_gap_ for CuB_11_N_12_ (87.5%). Ni and Cu nanocages exhibited higher sensitivity, while Fe and Co displayed stronger interactions but with prolonged recovery times. Ammar et al.^24^ analyzed Mn and Fe doping in B_12_N_12_ and the adsorption of CO, NO, and NH_3_. Doping reduced *E*_gap_ from 6.748 to 2.199 and 2.333 eV, enhancing adsorption and altering optical properties, such as shifting λ_max_ from 195 to 419 nm (Fe). Gases like NH_3_ induced changes in *E*_gap_ of up to +26.6%. Qadir et al.^25^ examined B_12_N_12_(Zn) and its interaction with various gases. The analysis revealed molecular stability and significant charge transfer with methodologies such as ELF and QTAIM elucidating interactions. The work advanced the design of sensing materials.

Hasanin et al.^26^ studied pure and Ni-decorated B_12_N_12_ for detecting CH_4_, H_2_S, and N_2_. The Ni@B_12_N_12_ system stood out, with an *E*_ads_ of −29.25 kcal/mol for H_2_S and substantial molecular orbital alterations, indicating promising sensitivity, particularly for H_2_S. In a previous work, we investigated the adsorption of N_2_O on pure and TM-modified B_12_N_12_ (Sc–Zn) nanocages using DFT-D3 and TD-DFT. While N_2_O showed weak interaction (physisorption) with pure B_12_N_12_ (*E*_ads_ = −0.14 eV), the Cu@B_12_N_12_ system demonstrated moderate chemical interaction (*E*_ads_ = −0.93 eV) and a high electronic sensitivity (Δgap = 72.3%) for gas detection. Furthermore, Cu@B_12_N_12_ showed promise for selective detection against interfering gases, also with a 50.4% variation in the work function.

Adalikwu et al.^27^ explored the adsorption of COCl_2_ on graphene/boron nitride (GP_Al_ and GP_BN_) heterostructures decorated with Zn, B, and O using DFT. They observed higher sensitivity in pristine systems, with the adsorption energy ranging from −2.15 to −11.65 eV. However, the decorated structures exhibited shorter recovery times, highlighting their potential as promising sensors. Hussain et al.^28^ in their theoretical work applied B_12_N_12_ nanocages decorated with copper (Cu@b_64_ and Cu@b_66_) for detection of COCl_2_. Their results revealed that phosgene binds more strongly to decorated BN nanocages in which greater charge transfer is also observed during adsorption. The interaction energies of COCl_2_ on copper-decorated B_12_N_12_ were −1.66 and −16.95 kJ/mol. Thus, the authors considered that decorated nanocages can be considered as potential candidates for applications in phosgene sensors. Xiong et al.^29^ published theoretical insights at the DFT level of the application of pure and metal-doped phytalocyanine (Pc) monolayers, such as manganese, aluminum, chromium, phosphorus, cobalt, and nickel, for the detection and monitoring of H_2_CO and COCl_2_ gases. The authors found that isolated 2D material (Pc) is not suitable for the adsorption of gases. However, metal-doped Pc can regulate the adsorption strength of the molecules due to moderate charge transfer and orbital hybridizations with gas molecules.

According to theoretical results and literature data, both B_12_N_12_ and Mn@b_64_ nanocages are possible and stable structures. Experimental studies have shown that it is possible to obtain B_12_N_12_ from boron nitride by top-down methods, such as plasma electrical discharge or plasma-assisted chemical vapor deposition.^21^ These techniques allowed nanocage samples to be obtained with high purity and morphology control. In addition, Oku and co-workers experimentally obtained other BN nanocages encapsulated with transition metals Fe, Y, Ag, and La.^30,31^ Based on the literature results and the synthesis methods already reported, the feasibility of synthesizing the Mn@b_64_ nanocage in the laboratory or even at scale is considered plausible for subsequent tests with toxic gases. Additionally, since their synthesis uses known methods and available instrumentation, nanocages also require low-cost precursors (absence of noble metals) and, furthermore, they can be supported, in small quantities, on matrices such as graphene^32^ in the production of sensors.

Despite the significant advances in this field, important challenges persist. Developing sensors capable of detecting phosgene under various temperature and humidity conditions, while ensuring high sensitivity and selectivity, alongside the demand for fast, cost-effective, and portable detection methods, remains a critical goal. In this context, the present study recognizes the lack of a systematic approach to the application of nanocages such as B_12_N_12_ modified with transition metals (TMs) as potential sensors for detecting COCl_2_ toxic gas. This work aims to investigate, through DFT calculations, how the different modifications (doped, decorated, and encapsulated) of B_12_N_12_ with TMs (Sc–Zn) can influence the adsorption, sensitivity, and selectivity of these nanomaterials for detecting phosgene gas in different environments.

## Computational Details

### Modeling and Optimization of Structures

**Figure 1:**
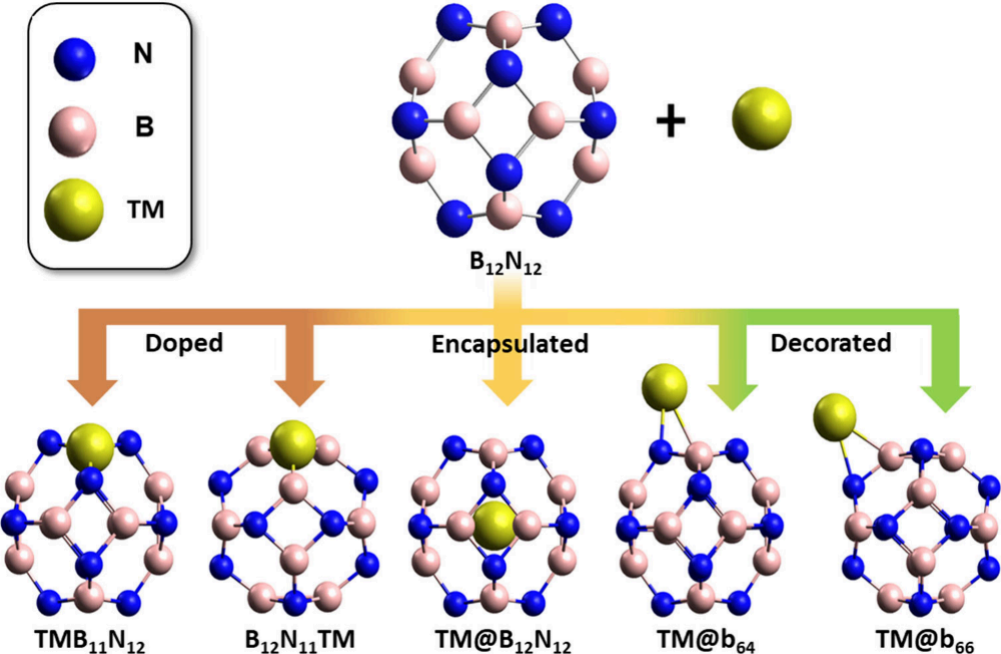
Illustration of the optimized structure of the B_12_N_12_ nanocage and representation of the doped (TM-B_11_N_12_ and B_12_N_11_TM), decorated (TM@b_64_ and TM@b_66_), and encapsulated (TM@B_12_N_12_) nanocages with the TM.

The B_12_N_12_ nanocage was modeled with high symmetry in a previous work^33^ and optimized at the DFT-D3 level^34^ with B3LYP and the 6-31G(d,p) basis set using the ORCA 5.0 package.^35^ B3LYP is a generalized gradient approximation (GGA) exchange functional, with 20% Hartree–Fock (HF), which has been successfully applied to deal with pure and modified B_12_N_12_ nanocages for diverse applications such as sensing, energy storage, catalysis, and drug delivery.^36,37^ The version of Grimme D3 scattering^34^ describes the long-range electronic correlations responsible for van der Waals interactions. The B_12_N_12_ nanocage was modified with the 10 first-row transition metals (TM = Sc–Zn) in five configurations as shown in Figure 1. Modification configurations of the B_12_N_12_ with TM were as follows:

- doped in B, by replacing a B atom with a TM (TM-B_11_N_12_);
- doped in N, by replacing a N atom with a TM (B_12_N_11_TM);
- decorated b_64_, with the positioning of the TM external to B_12_N_12_, on one of the bonds that divides a hexagonal ring and a tetragonal ring (TM@b_64_);
- decorated b_66_, with the positioning of the TM external to B_12_N_12_, above one of the connections that divides two hexagonal rings (TM@b_66_);
- encapsulated, with the TM positioned inside the cage (TM@B_12_N_12_).

The decorated positions atop the tetragonal or hexagonal rings and atop the B or N atoms were not investigated here, as they are less stable than positions b_64_ and b_66_, as already reported in the literature.^28,38,39^ These neutral structures, in their most stable spin states (Figure S1), were used in this work to study the adsorption of the toxic gas COCl_2_ and interferents. Here, the adsorption of phosgene on the surfaces of pure and modified B_12_N_12_ (TM-B_12_N_12_) was investigated using the same level of theory. Vibrational frequencies were also calculated to guarantee the absence of imaginary (negative) frequencies and ensure that the optimized structures are not transition states (TS). The criteria of energy convergence, RMS gradient, RMS displacement, maximum gradient, and maximum displacement were used in the calculations: 5 × 10^–6^ Hartree, 1 × 10^–4^ Hartree/Bohr, 2 × 10^–3^ Bohr, 3 × 10^–4^ Hartree/Bohr, and 4 × 10^–3^ Bohr, respectively.

### Structural and Energy Analysis

To investigate the degree of interaction between nanocages and TMs, the cage/TM interaction energy (*E*_int_) was calculated by subtracting from the energy of the nanocage with the TM (*E*_(TM–nanocage)_) the energies of the nanocage (*E*_(nanocage)_) and the TM (*E*_(TM)_, in a bulk metal with a more stable spin state). This concept has been widely used in the literature^38−40^ and can be applied according to eq 1:
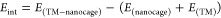
1

The difference between the energy level of the lowest unoccupied molecular orbital (LUMO, *E*_L_) and the highest occupied molecular orbital (HOMO, *E*_H_) establishes the energy gap of each system (*E*_gap_). The percentage variation in the gap of each system before and after the adsorption of COCl_2_ gas (Δgap) can be related to the electronic sensitivity of the nanocage to the gas, and here it was calculated with eq 2:
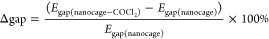
2where *E*_gap(nanocage–COCl_2_)_ is the energy gap of B_12_N_12_–COCl_2_ or TM-B_12_N_12_–COCl_2_ and *E*_gap(nanocage)_ is the gap of pure B_12_N_12_ or modified nanocage TM-B_12_N_12_.

Quantum mechanical descriptors such as chemical potential (μ), global hardness (η), and electrophilicity (ω) were calculated using eqs 3–5:^41,42^
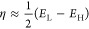
3
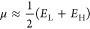
4
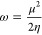
5where *E*_L_ and *E*_H_ are the LUMO and HOMO energies, respectively.

Based on the principles of maximum hardness (η)^43^ and minimum electrophilicity (ω),^44^ it is clear that systems with greater chemical hardness and lower electrophilicity are more stable. Thus, electrophilicity (ω) is calculated and used as a parameter to determine the stability of systems after the interaction with the gas. To be careful, the system stability study also uses Gibbs free energy analysis (Δ*G*_ads_), which is calculated according to eq 6:
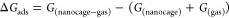
6where *G*_(nanocage–gas)_ is the free energy after adsorption of COCl_2_ gas, *G*_(nanocage)_ is the free energy of the isolated cage, and *G*_(ga*s*)_ is the Gibbs free energy of COCl_2_.

### Application Analysis as a Material for Sensors

To adequately investigate the potential application of nanocages as a material for chemical sensors for detecting COCl_2_ gas, the following terms were calculated: the electrical conductivity of the system (σ), which is related to the electronic changes of the nanocage upon adsorption of COCl_2_; the adsorption energy of the gas on the nanocage surface (*E*_ads_); the electric current density (*j*), related to the work function (Φ); the recovery time of the sensor (τ); the sensitivity (*S*) and selectivity coefficient (κ) against other gases; and the UV–vis spectrum, which deals with the optical sensitivity of the system to the gas.

The electrical conductivity (σ) of pure B_12_N_12_ nanocages and those modified with TMs before and after the adsorption of COCl_2_ gas was calculated using eq 7:^45^
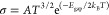
7where *A* (electron m^–3^ K^–3/2^) is a constant, *T* is the thermodynamic temperature (kelvin), *E*_gap_ is the energy gap, and *k*_B_ is the Boltzmann constant (8.62 × 10^–5^ eV K^–1^). The adsorption energy (*E*_ads_) was calculated using eq 8:
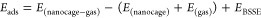
8where *E*_(nanocage–gas)_ is the energy of the system formed with COCl_2_ gas adsorbed on B_12_N_12_ or TM-B_12_N_12_, *E*_(nanocage)_ is the energy of pure B_12_N_12_ or TM-B_12_N_12_, *E*_(gas)_ is the energy of the COCl_2_ molecule, and *E*_BSSE_ is the base superposition error energy (BSSE).

Based on electronic sensitivity and adsorption energy analyses, the best nanocages for application in gas sensing were isolated and then a parameter considered crucial for the study of sensors was determined, the recovery time (τ), which is exponentially related with the *E*_ads_ of the system, as shown in eq 9:^46^
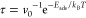
9where *v*_0_ is the attempt frequency (5.2 × 10^14^*v*_0_ s^–1^).^47^ In turn, the variation in the electrical current density in the sensor (*j*) can be experimentally associated with the value of the work function (Φ) before and after the interaction of the nanocage with the molecule, according to eq 10:^48,49^
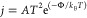
10where *A* is the Richardson–Dushman constant (theoretical value of 120.1 A m^–2^ K^–2^)^50^ and others have already been defined previously. The aim was to evaluate the applicability of pure B_12_N_12_ and the system with the best result found as a work function sensor material (Φ). The work function here is determined as a function of the frontier orbitals, as shown in eq 11, and the function variation (ΔΦ), before and after gas adsorption, determines the potential application of the nanocage as a sensor, being calculated here, as shown in eqs 11 and 12:
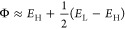
11
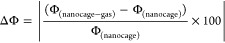
12

The system that presented the best result for detecting COCl_2_ was also subjected to interaction with water and other gases considered interfering (H_2_, CH_4_, CO_2_, NH_3_, N_2_, CO, H_2_S, and N_2_O), to investigate its selectivity for COCl_2_ adsorption against interferents. Sensitivity was evaluated for both the conductometric variation (*S_σ_*) and the work function (*S_j_*), calculating the sensor response and selectivity coefficient (κ), using eqs 13–15:^50^
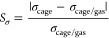
13
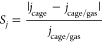
14
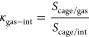
15where σ_cage/gas_ and *σ*_cage_ are the conductivities of the cage with and without the adsorbed gas, respectively. Similarly, *j*_cage/gas_ and *j*_cage_ are the current densities of the cage with and without the adsorbed gas, respectively. To investigate the application of the material as an optical sensor for COCl_2_ gas detection, the time-dependent density functional theory (TD-DFT) method is employed with 100 roots for the analysis of transition states and calculation of the UV–vis spectra of the structures before and after adsorption. The graphs were plotted using MultiWfn.^51^ The stability for the best system found after COCl_2_ adsorption is evaluated through a 250 ps quantum molecular dynamics (MD) with an integration interval of 2 fs, in which the force calculation uses the GFN-1 Hamiltonian implemented in the xTB software.^52^ The influence of humidity was also analyzed with MD, with a “box” with 40 water molecules, containing COCl_2_ and the best-responding nanocage, in order to test the dynamic behavior of the cage/gas interaction in an aqueous medium and indirectly the efficiency of the sensor in high humidity.

## Results and Discussion

### TM-Modified B_12_N_12_ Nanocages

The B_12_N_12_ nanocage was optimized in isolation and then modified with a TM in five different configurations (TM-B_11_N_12_, B_12_N_11_TM, TM@b_64_, TM@b_66_, and TM@B_12_N_12_), as illustrated in Figure 1. The positions decorated on the tetragonal or hexagonal rings and on the B or N atoms were not considered here, as they are less stable than positions b_64_ and b_66_, as already reported in the literature.^28,38,39^ Geometry optimization shows a very stable BN cluster with high symmetry and zero dipole moment, where its nitrogen and boron atoms occupy equivalent structural vertices. B_12_N_12_ is made up of six tetragonal (four-membered) and eight hexagonal (six-membered) rings. The cluster has two bond lengths, so that the length of the B–N bond varies depending on its position, either between a tetragonal ring and a hexagonal ring (b_64_), whose length is 1.48 Å, or between two hexagonal rings (b_66_), measuring 1.43 Å. These parameters correspond to literature data.^53−55^ More comprehensive work has investigated the influence of the interactions of 3d metals on the increase in the the properties of the systems.^38,39,55,56^ TM-B_12_N_12_ interactions and their structural, electronic, and optical properties have been discussed in previous works^28,44^ dedicated to DFT analysis of nanocage modification with first-row TMs.

After modification of B_12_N_12_ with the TMs, the cage/metal interaction energy (*E*_int_) was calculated, which provides information about the degree of chemical affinity of the nanocage with the metal, given the five configurations of interaction tested. The calculated values of *E*_int_ are listed in Table 1. They indicate that the best and worst interactions occur between B_12_N_12_ and the metals Sc and Zn, respectively: ScB_11_N_12_ (*E*_int_ = −8.65 eV) and Zn@B_12_N_12_ (*E*_int_ = 13.40 eV). In general, encapsulated systems showed lower (less negative) interaction energy values, followed by decorated systems, and doped systems showed higher cage/metal interaction values when comparing the different interaction configurations for each TM. This suggests that B_12_N_12_ is sensitive to different interaction metal configurations. Similar behavior is observed in the work of Abbasi and collaborators for decorated systems.^39^ It is also observed that the interaction of TM with B_12_N_12_ becomes less intense with an increase in the TM atomic number (Sc to Zn). Finally, we observed that the modification with TM alters the energy gap, charge transfer, and reactivity of the nanocage, as shown below, just as the doping of graphene (Gr) with N (N-Gr), in the study reported by Jin,^57^ increases the dispersion and interaction with TM clusters, altering the growth mechanism and charge transfer.

**Table 1:** Interaction Energy Values (*E*_int_, in electronvolts) for the TM-B_12_N_12_ Nanocages

	TMB_11_N_12_	B_12_N_11_TM	TM@b_64_	TM@b_66_	TM@B_12_N_12_
Sc	–8.65	–1.61	–0.66	1.28	3.55
Ti	–7.95	–1.88	–0.36	1.96	5.01
V	–6.90	–1.40	–0.12	2.27	5.98
Cr	–6.91	–3.01	–1.55	0.76	5.82
Mn	–4.50	–0.29	2.81	3.02	8.60
Fe	–3.42	–0.41	1.71	3.54	8.02
Co	–3.11	–0.73	3.92	4.21	6.81
Ni	–2.89	–0.33	1.63	4.26	7.37
Cu	0.42	2.26	5.86	6.06	8.90
Zn	–0.08	2.98	5.92	5.92	13.40

Initially, as we have shown in previous works,^33^ the singlet multiplicities for Zn metal, doublet multiplicities for Sc and Cu, singlet and triplet multiplicities for Ti and Ni, doublet and quartet multiplicities for V and Co, and singlet, triplet, and quintet multiplicities for Cr and Fe were tested, as well as doublet, quartet, and sextet multiplicities for Mn. The TM spin multiplicity for which each nanocage was most stable is shown in Figure S1, and only these multiplicities are considered for the subsequent analyses. Additionally, α (α-spin up) and β (β-spin down) orbitals were considered for open shell systems.

The energy gap (*E*_gap_) of the isolated B_12_N_12_ and the TM-modified nanocages were estimated from the electronic properties, considering the most stable multiplicity of each system. The *E*_gap_ and the respective frontier orbitals (HOMO and LUMO) are shown in Table S1. The energy gap of B_12_N_12_ (*E*_gap_ = 6.88 eV) is effectively reduced when it is modified with any TM in any configuration.^24,29,49^ These characteristics reveal that the presence of a metal makes the nanocage more conductive and reactive. The largest reduction in the *E*_gap_ of the cage occurs in the CuB_11_N_12_ nanocage (*E*_gap_ = 2.05 eV), and the smallest reduction is observed for VB_11_N_12_ (*E*_gap_ = 4.98 eV). The reduction in the gap after the interaction occurs due to the destabilization of LUMO, which presents a greater contribution from the metal, which is more electropositive compared to B and N, and also due to strong HOMO stabilization promoted by the presence of TM.

### Study of COCl_2_ Gas Adsorption

**Figure 2:**
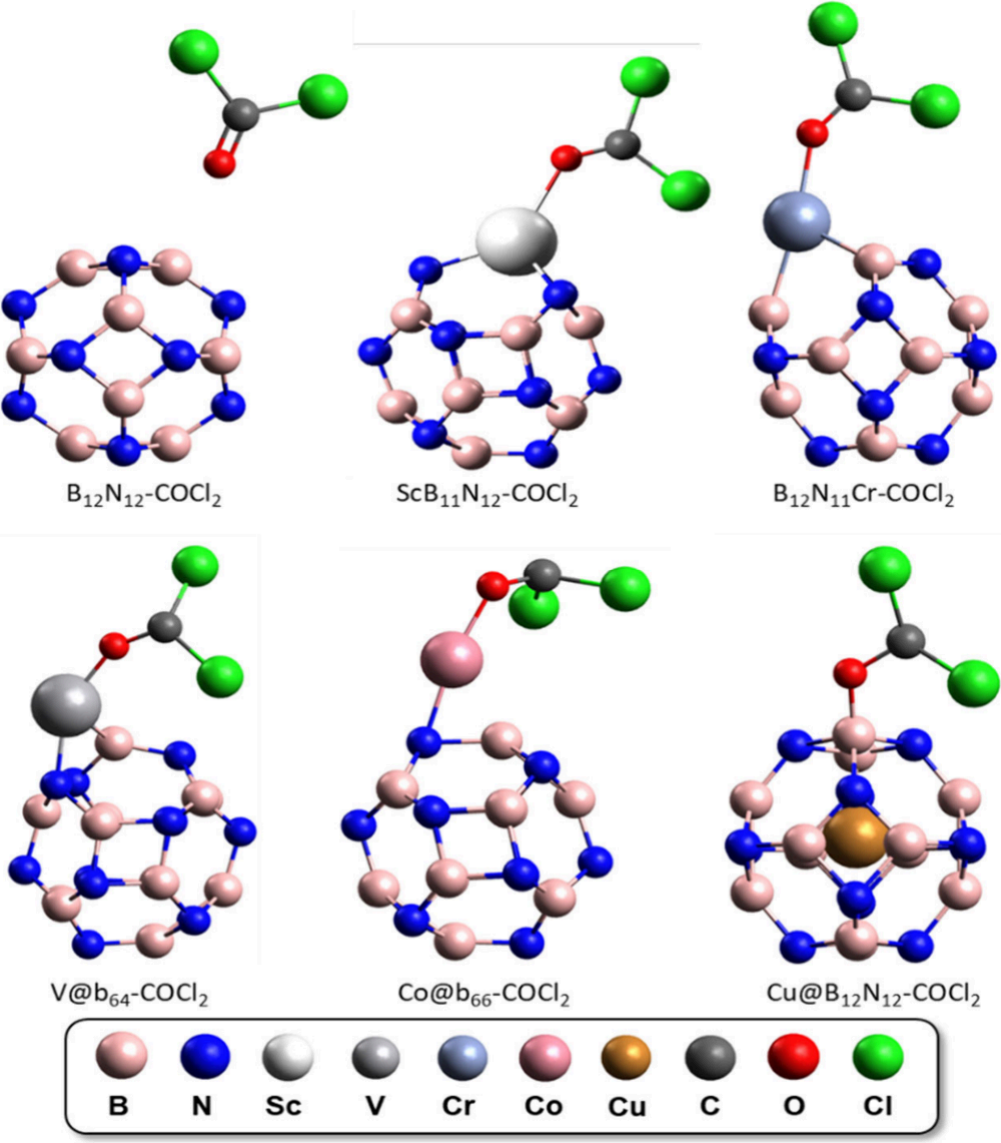
Examples of optimized COCl_2_ gas adsorption structures on the surface of isolated B_12_N_12_ and TM-modified nanocages (doped, decorated, and encapsulated).

The study of adsorption of COCl_2_ gas on the surface of pure and modified B_12_N_12_ nanocages was performed with the B_12_N_12_-COCl_2_, ScB_11_N_12_-COCl_2_, B_12_N_11_Cr-COCl_2_, V@b_64_-COCl_2_, Co@b_66_-COCl_2_, and Cu@B_12_N_12_-COCl_2_ systems in Figure 2, where it is observed that the COCl_2_ molecule interacts through its O atom with B of B_12_N_12_ isolated and encapsulated and with the TM of doped and decorated nanocages.^28,58^ The images show that phosgene does not establish a bond with B_12_N_12_, remaining farther from the cage (*d* = 2.513 Å), which is related to a weak interaction.

**Figure 3:**
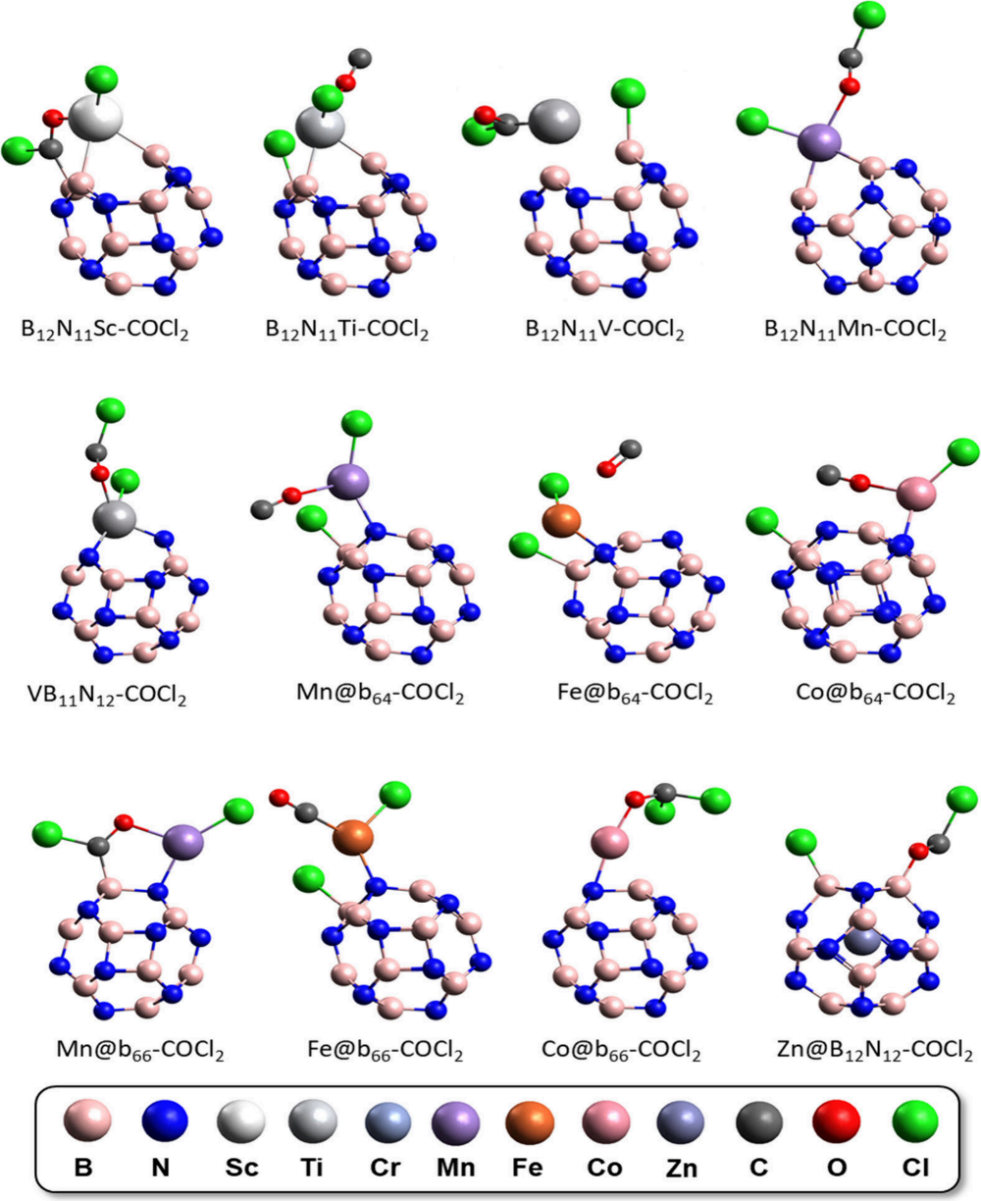
Optimized structures of TM-modified B_12_N_12_ nanocages on which the COCl_2_ molecule undergoes dissociation.

The relaxation of the geometry of the adsorption structures showed that in some cases the gas/cage interaction does not occur via simple connection of the cage with the oxygen in the gas, as illustrated in Figure 2. However, all of the different forms of adsorption of COCl_2_ in the cages are shown in Figure 3 (gas dissociation adsorption). It is possible to observe the dissociation of the COCl_2_ gas molecule when it interacts with the B_12_N_11_Sc, B_12_N_11_Ti, B_12_N_11_V, B_12_N_11_Mn, VB_11_N_12_, Mn@b_64_, Fe@b_64_, Co@b_64_, Mn@b_66_, Fe@b_66_, Co@b_66_, and Zn@B_12_N_12_ nanocages. These adsorption systems are stable, as will be demonstrated below by the analysis of the free energy of adsorption, and in these, the separation of one or two chlorine atoms from the structure is observed, with formation of COCl + Cl or CO + 2Cl. The dissociation products of COCl_2_ (CO, Cl radicals, and Cl_2_) exhibit different levels of toxicity: CO reduces blood oxygenation, Cl radicals are highly reactive, and Cl_2_ is corrosive and irritating.^59^ However, the number of dissociated molecules during detection is minimal, causing no significant environmental impact. Dissociation on the nanocage surface is beneficial for biosensors, as the reactive products enhance interaction with receptors, improving sensitivity and detection efficiency. Nonetheless, stability tests for the nanocages should be conducted to evaluate potential material degradation caused by the interaction with these toxic products.

This behavior is also reported in some works found in the literature, such as in the study by Lizardo-Huerta and collaborators, who investigated the decomposition of phosgene and diphosgene,^60^ and in the work by Esrafili and collaborators,^61^ who studied the reduction of NO on the surface of BN nanocages. One can also observe the decomposition of N_2_O into N_2_ occurring on the B_12_N_12_ surface in the work of Baei et al.^62^ and in our previous work,^63^ in which we promoted the adsorption of N_2_O in B_12_N_12_ nanocages modified with a TM for selective detection analysis of N_2_O gas.

Under these conditions, the frontier orbitals (HOMO and LUMO) of the structures formed from the adsorption of COCl_2_ gas were collected and the energy gap (*E*_gap_) was calculated for these systems. The values are presented in Table S2. It was observed that the Fe@b_64_-COCl_2_ system presents a larger HOMO–LUMO gap after gas adsorption (*E*_gap_ = 6.19 eV) and that the system with Sc-encapsulated Sc@B_12_N_12_-COCl_2_ has the smallest gap in the series (*E*_gap_ = 0.95 eV). It is also possible to observe that decorated systems have higher *E*_gap_ values when compared with doped and encapsulated systems. However, pure B_12_N_12_ continues to present a higher *E*_gap_ than the modified systems^55,58^ even after interaction with the gas, except for the Fe@b_64_-COCl_2_ system. This indicates that the modified nanocages suffer greater band gap variation when interacting with the gas, and therefore, they are more electronically sensitive to the presence of phosgene.

**Figure 4:**
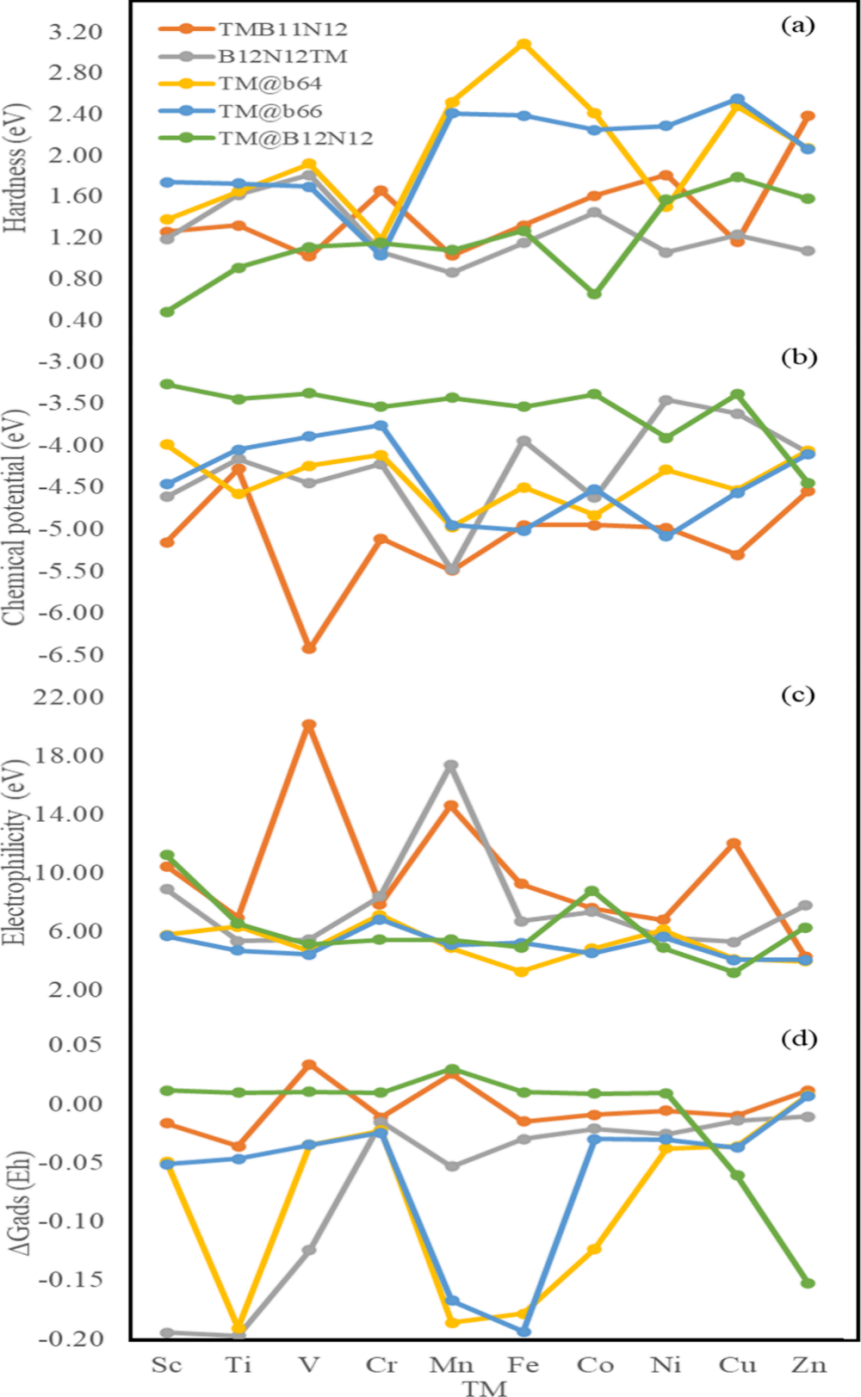
(a) Hardness η, (b) chemical potential μ, (c) electrophilicity ω, and (d) variation in the Gibbs free energy Δ*G*_ads_ of COCl_2_ gas adsorption structures in modified B_12_N_12_ nanocages.

The quantum descriptors for isolated B_12_N_12_ and TM-modified nanocages were presented and extensively discussed in a previous work.^33^ The calculation of the quantum descriptors for the systems after gas adsorption allows us to discuss possible electronic or stability changes caused by the interaction with the phosgene molecule. To analyze the stability of the adsorption systems, quantum descriptors such as chemical potential (μ), global hardness (η), and electrophilicity (ω) were calculated here based on eqs 3–5^28,39,49,63,64^ and plotted on the graphs in Figure 4, in addition to the Gibbs free energy values (Δ*G*_ads_).^49,64,65^ The chemical hardness values show that the ScB_11_N_12_ system has the lowest hardness of the entire series, while the adsorption of COCl_2_ on Fe@b_64_ showed a higher chemical hardness value and therefore a lower reactivity, which is in accordance with its large recorded energy gap. The calculated chemical potential for the interaction between the modified nanocages and phosgene shows lower values for the encapsulated systems and higher values for the systems doped with TM instead of boron (TM-B_11_N_12_). The electrophilicity of the systems is shown in Figure 4, which indicates greater electrophilic character for the VB_11_N_12_-COCl_2_ and B_12_N_11_Mn-COCl_2_ systems and lower electrophilicity for the complexes generated by Mn@b_64_-COCl_2_ interactions, Fe@b_64_-COCl_2_ and Cu@B_12_N_12_-COCl_2_, these systems being more stable.

The variation in the Gibbs free energy is considered before and after gas adsorption and provides information about the adsorption stability. *ΔG*_ads_ presents positive values for systems with encapsulated TMs, indicating the nonspontaneity of the process, with the exception of adsorption systems with encapsulated Cu and Zn, which show favorable interaction, corroborated by the high level of transfer of electron density to the gas, as shown below. This different behavior for Cu and Zn in first-row metal complexes has been reported in several studies in the literature, in terms of binding energy, charge transfer, and electronic properties.^33,38,56,66^ These factors justify the fact that Cu and Zn exhibit different complexation behaviors. In particular, the combination of filled d orbitals, a higher electronegativity, a smaller radius, and a weak interaction with the cage explains why Cu and Zn nanocages spontaneously adsorb COCl_2_ gas.

On the other hand, the interactions between the gas and the B_12_N_11_Sc, B_12_N_11_Ti, Ti@b_64_, Mn@b_64_, Fe@b_64_, Mn@b_66_, and Fe@b_66_ nanocages present the highest (most negative) stability values of the series (*ΔG*_ads_ = −0.19, −0.2, −0.19, −0.19, −0.18, −0.17, and −0.19 eV, respectively). The greater stability of Fe@b_64_ is consistent with its high chemical hardness discussed previously, and the high stability of Mn@b_64_ is related to its low electrophilicity.

**Figure 5:**
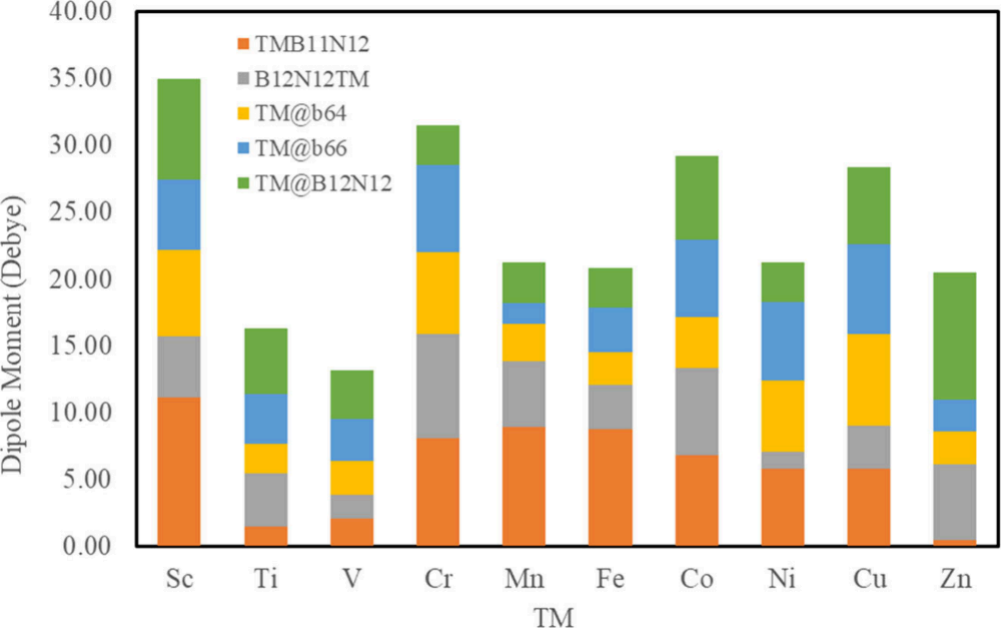
Dipole moments (DMs) calculated for B_12_N_12_ nanocages modified with a TM after the adsorption of COCl_2_ gas.

The molecular dipole moment (DM) was calculated for the adsorption systems (Figure 5), to evaluate the charge distribution in them. Based on the data obtained, it is possible to observe that systems modified with Ti and V present lower DM values compared with systems with other TMs, regardless of the metal configuration. The DM depends on the number of charges as well as their separation. Less intense loads are responsible for the low dipole moment.^28^ The ZnB_11_N_12_-COCl_2_ system has the lowest dipole value in the series (DM = 0.48 D), despite the complexes being doped with a TM instead of B, in general, presenting the highest DM values, i.e., greater separation of charges in the complex. On the other hand, the ScB_11_N_12_-COCl_2_ system presents the highest dipole moment value (DM = 11.16 D), which can be related to the lower hardness presented by the system.

**Figure 6:**
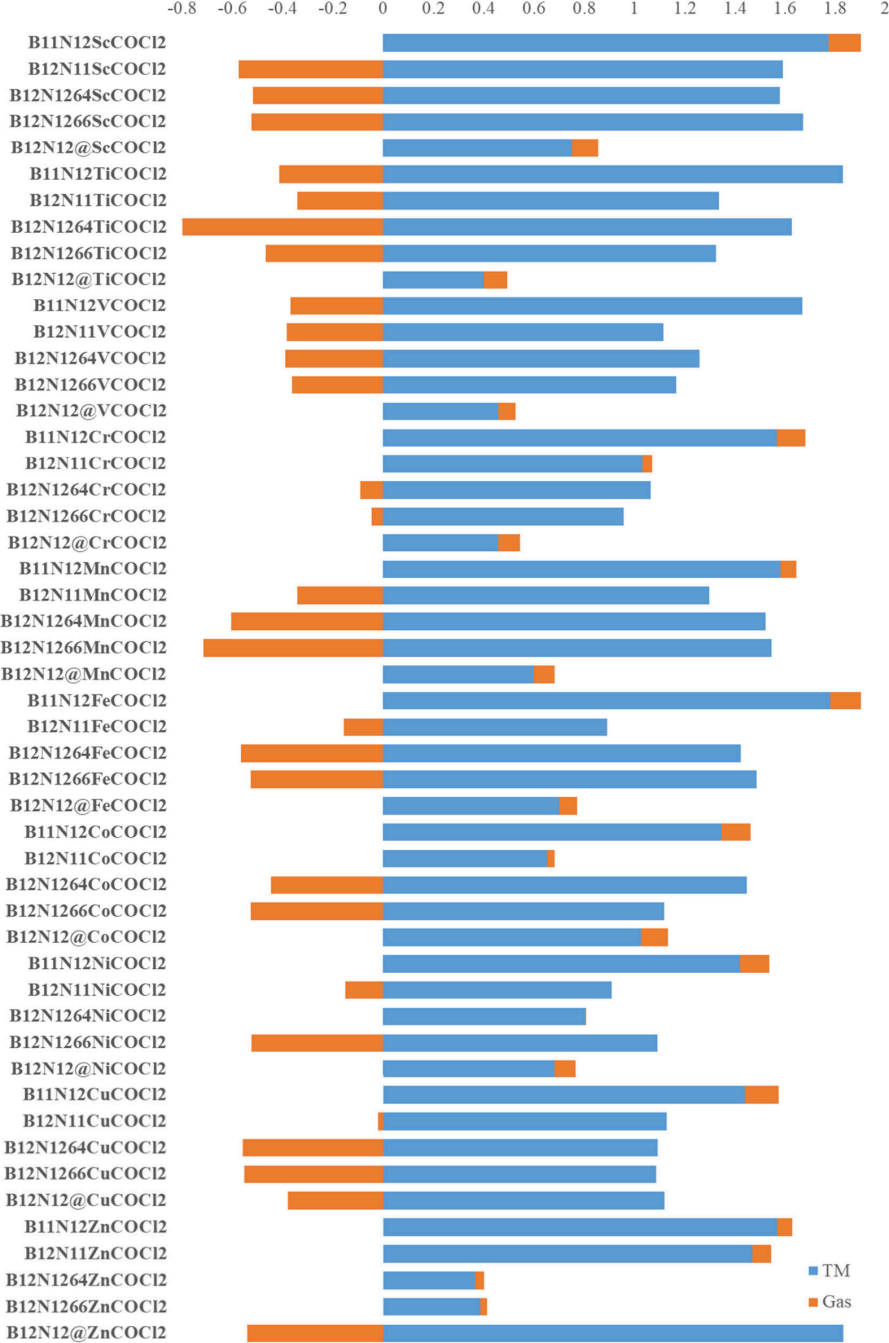
Distribution of NPA charges (*Q*) on COCl_2_ gas and on the TM of the B_12_N_12_ nanocages modified after the adsorption of COCl_2_ gas.

Considering that the isolated phosgene molecule has a residual charge equal to zero, the charge acquired by the molecule after the interaction can be related to the transfer of electron density from the cage to the gas (negative value) or from the gas to the cage (positive charge). In Figure 6, the NPA charge values of COCl_2_ and TM are presented according to NBO (natural bond orbital) analysis. Phosgene gas has a charge of 0.502 when interacting with B_12_N_12_ alone. It is possible to observe that metals acquire a negative charge in encapsulated systems, except for Cu and Zn, while in other systems, the TM acquires a positive charge. This suggests that the metal loses electronic density in the adsorption process. In relation to the phosgene molecule, it appears that it acquires a negative charge, which allows it to be related to the behavior of a Lewis acid, receiving electrons from the nanocage. However, in some cases, the gas gives up electrons to the cage, becoming positively charged, as observed in encapsulated systems from Sc to Ni and in systems doped with a TM instead of B (except for TiB_11_N_12_ and VB_11_N_12_). The lowest level of charge transfer occurs from COCl_2_ to Ni@b_64_ (−0.070), while the highest electron density recorded is transferred from the phosgene to the Ti@b_64_ nanocage (−1.274). In general, COCl_2_ tends to withdraw electrons from the cage and the TM, a behavior justified by the presence of very electronegative elements in the molecule, such as chlorine.

### Study of Application in Sensors for COCl_2_

The application of nanostructures in sensing devices requires the observation of parameters such as the variation of the energy gap (Δgap), adsorption energy (*E*_ads_), recovery time (τ),^31^ sensitivity (*S*) and variation of work function (ΔΦ) and optical properties, for application in optical sensors. Thus, to investigate the application of modified B_12_N_12_ in the phosgene sensor, these parameters were calculated and are listed in Table 2. The electronic sensitivity of the material to the gas is related to the variation of the electrical conductivity of the sensor, according to eq 7, which depends on the variation of *E*_gap_, before and after adsorption.

**Table 2:** Variations of the Gap (Δgap) and Work Function (ΔΦ), Adsorption Energies (*E*_ads_), and Recovery Times (τ) for TM-B_12_N_12_-COCl_2_ Systems

system	Δgap (%)	ΔΦ (%)	*E*_ads_ (eV)	τ	system	Δgap (%)	ΔΦ (%)	*E*_ads_ (eV)	τ
B_12_N_12_-COCl_2_	21.80	12.65	–0.11	722.45 fs					
ScB_11_N_12_-COCl_2_	40.61	11.47	–0.55	19.68 μs	FeB_11_N_12_-COCl_2_	40.81	10.49	–0.72	14.68 ms
B_12_N_11_Sc-COCl_2_	8.43	22.28	–0.37	17.88 ns	B_12_N_11_Fe-COCl_2_	27.22	4.23	–1.49	4.18 × 10^7^ h
Sc@b_64_-COCl_2_	10.44	27.48	–0.12	1.07 ps	Fe@b_64_-COCl_2_	124.28	22.28	–0.99	8.94 min
Sc@b_66_-COCl_2_	36.47	23.20	–0.81	487.08 ms	Fe@b_66_-COCl_2_	47.53	49.11	–0.84	1.57 s
Sc@B_12_N_12_-COCl_2_	56.62	12.37	–1.1	10.76 h	Fe@B_12_N_12_-COCl_2_	36.59	15.69	–0.19	16.24 ps
TiB_11_N_12_-COCl_2_	14.33	12.04	–0.91	23.85 s	CoB_11_N_12_-COCl_2_	17.27	3.32	–0.12	1.07 ps
B_12_N_11_Ti-COCl_2_	10.62	2.46	–1.42	2.74 × 10^6^ h	B_12_N_11_Co-COCl_2_	27.02	8.71	–1.34	1.22 × 10^5^ h
Ti@b_64_-COCl_2_	28.91	33.92	–1.04	1.04 h	Co@b_64_-COCl_2_	55.66	42.06	–1	13.19 min
Ti@b_66_-COCl_2_	28.15	10.96	–1.06	2.27 h	Co@b_66_-COCl_2_	26.40	1.35	–0.94	1.28 min
Ti@B_12_N_12_-COCl_2_	40.46	9.87	–0.17	7.46 ps	Co@B_12_N_12_-COCl_2_	58.28	21.07	–1.51	9.12 × 10^7^ h
VB_11_N_12_-COCl_2_	59.04	38.96	0.79	–	NiB_11_N_12_-COCl_2_	8.33	6.74	–0.61	203.21 μs
B_12_N_11_V-COCl_2_	18.75	10.97	–1.4	1.26 × 10^6^ h	B_12_N_11_Ni-COCl_2_	16.21	5.46	–1.24	2497.77 h
V@b_64_-COCl_2_	8.19	9.28	–1.3	2.57 × 10^4^ h	Ni@b_64_-COCl_2_	5.97	3.87	–1.97	5.41 × 10^15^ h
V@b_66_-COCl_2_	23.72	7.16	–1.45	8.83 × 10^6^ h	Ni@b_66_-COCl_2_	20.53	11.89	–1.02	28.71 min
V@B_12_N_12_-COCl_2_	34.32	12.67	–0.5	2.81 μs	Ni@B_12_N_12_-COCl_2_	35.73	14.66	–0.27	365.2 ps
CrB_11_N_12_-COCl_2_	32.93	9.42	–0.59	93.32 μs	CuB_11_N_12_-COCl_2_	13.17	9.56	–0.65	963.55 μs
B_12_N_11_Cr-COCl_2_	32.05	5.50	–1.01	19.46 min	B_12_N_11_Cu-COCl_2_	10.87	4.23	–0.15	3.43 ps
Cr@b_64_-COCl_2_	27.83	2.49	–1.18	241.91 h	Cu@b_64_-COCl_2_	18.42	2.79	–1.09	7.29 h
Cr@b_66_-COCl_2_	23.42	6.82	–1.24	2497.77 h	Cu@b_66_-COCl_2_	21.43	1.94	–1.05	1.54 h
Cr@B_12_N_12_-COCl_2_	34.84	9.94	–0.07	152.36 fs	Cu@B_12_N_12_-COCl_2_	31.73	24.18	–1.3	2.57 × 10^4^ h
MnB_11_N_12_-COCl_2_	54.53	11.59	0.4	–	ZnB_11_N_12_-COCl_2_	1.70	4.22	–0.51	4.15 μs
B_12_N_11_Mn-COCl_2_	37.32	65.06	–0.79	223.68 ms	B_12_N_11_Zn-COCl_2_	27.21	0.97	–0.92	35.19 s
Mn@b_64_-COCl_2_	69.13	24.87	–0.48	1.29 μs	Zn@b_64_-COCl_2_	8.19	1.22	–0.04	47.42 fs
Mn@b_66_-COCl_2_	48.46	43.90	–0.29	795.24 ps	Zn@b_66_-COCl_2_	8.63	0.24	–0.03	32.13 fs
Mn@B_12_N_12_-COCl_2_	49.05	6.19	–5.86	2.90 × 10^81^ h	Zn@B_12_N_12_-COCl_2_	45.16	63.60	–0.59	93.32 μs

The results show that the Δgap of isolated B_12_N_12_ and B_12_N_12_ after gas adsorption is 21.8%, which is in agreement with data reported in the literature^28^ and is a better result than the value of the modified nanocages (36%). The ZnB_11_N_12_ nanocage presents the lowest sensitivity of the entire series (Δgap = 1.7%). On the other hand, six nanocages presented Δgap values of higher than 50% for gas detection, such as Co@b_64_ (55.56%), Sc@B_12_N_12_ (56.62%), Co@B_12_N_12_ (58.28%), VB_11_N_12_ (59.04%), Mn@b_64_ (69.13%), and Fe@b_64_ (124.28%). Hussain et al.^28^ decorated B_12_N_12_ with copper to detect COCl_2_ and reported Δgap and *E*_ads_ values of 25.2% and 8.3% and −0.02 and −0.18 eV for the Cu@b_64_ and Cu@b_66_ systems, respectively. In contrast, our data show a Δgap for Cu@b_64_ that is larger than that for Cu@b_66_. However, we found higher *E*_ads_ values for both systems (−1.09 and −1.5 eV, respectively). This difference in the values found may be related to the use of D3 and BSSE in our calculations, since the non-use of the dispersion factor (D3) in the calculations tends to generate underestimated *E*_ads_ values, as shown in the work of Jin et al.^57^ Finally, the Mn@b_64_ and Fe@b_64_ systems stand out with superior sensitivity and great potential for application in COCl_2_ toxic gas sensors.

The work function is related to the variation of the electric current density (*j*) of the sensor (eq 10) and indicates the potential application of the material in devices of this nature. The work function results are presented for B_12_N_12_-COCl_2_ (ΔΦ = 12.65%) and the best value for B_12_N_11_Mn (ΔΦ = 65.06%), with Mn@b_64_-COCl_2_ presenting a ΔΦ of 25.87%. These data indicate that the B_12_N_11_Mn nanocage is the best candidate for applications in work function-type sensors for phosgene detection. The intensity of the adsorbate interaction on the substrate surface is described by *E*_ads_. Negative *E*_ads_ values characterize more stable interactions. However, according to the literature, values below −0.3 eV (less negative) characterize purely physical interactions.^46^ The *E*_ads_ shows that isolated B_12_N_12_ weakly adsorbs phosgene (*E*_ads_ = −0.11 eV), with a physisorption-type interaction, and the weak interaction observed justifies the low level of charge transfer recorded. Some of the modified nanocages presented positive *E*_ads_ values when adsorbing phosgene, indicating unstable interactions, as observed for VB_11_N_12_-COC_l2_ (*E*_ads_ = 0.79 eV) and MnB_11_N_12_-COCl_2_ (*E*_ads_ = 0.40 eV).

The recovery time (τ) is an essential parameter to assess the feasibility of applications in sensors. Desorption, being the inverse of adsorption, presents its energy barrier (*E*_atv_) that is equal to the value of the adsorption energy (*E*_ads_). Thus, systems with a very high adsorption energy present a long recovery time. Peng et al.,^67^ in a study with carbon nanotubes as NO_2_ gas sensors, demonstrated that, with a range of *E*_ads_ values between −0.34 and −0.79 eV, the recovery time varies from 5 μs to 16 s, while an *E*_ads_ of greater than −1 eV results in recovery times of greater than 12 h.

Analyzing the *E*_ads_ and recovery time of the nanocages (Table 2) with good electronic sensitivity above 50%, we observed that the Sc@B_12_N_12_, Fe@b_64_, Co@b_64_, and Co@B_12_N_12_ systems presented very high *E*_ads_ values (−1.1, −0.99, −1, and −1.51 eV, respectively), indicating a very intense gas adsorption and consequently a very long recovery time (τ = 11 h, 9 min, 13 min, and >12 h, respectively), making their use as fast response sensors for phosgene unfeasible. A similar characteristic was reported by Deji et al.;^19^ when adsorbing phosgene on graphene nanoribbons doped with B and P, they reported an *E*_ads_ in the range of −4.4 to −12.2 eV for the doped systems. In contrast, the VB_11_N_12_ system showed weak adsorption and a positive *E*_ads_ value (0.79 eV), resulting in spontaneous desorption of the gas. However, the Mn@b_64_ nanocage showed a moderate *E*_ads_ (−0.48 eV), with an excellent recovery time of 1.29 μs. Therefore, recording a moderate chemical interaction prevents spontaneous desorption of the gas at room temperature and a recovery time that maintains the availability of the sensor for consecutive detections. Thus, the Mn@b_64_ nanocage shows itself to be a promising material for applications as a fast-response and highly sensitive sensor for COCl_2_.

**Figure 7:**
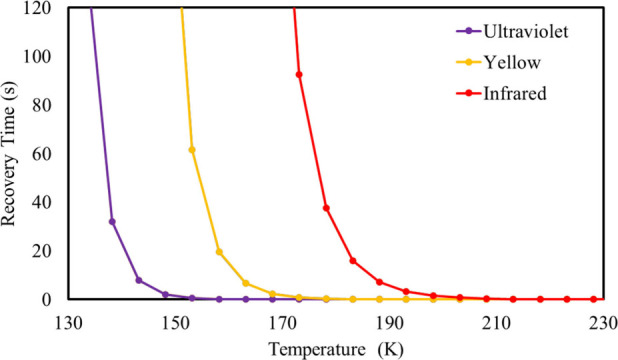
Variation of recovery time for the Mn@b_64_-COCl_2_ system according to the temperature and different trial frequencies.

To extrapolate the analysis of variation in the recovery time of the Mn@b_64_-COCl_2_ system, different trial frequencies were used in the calculations (1.0 × 10^12^ s^–1^ for the infrared frequency, 5.2 × 10^14^ s^–1^ for light yellow radiation, and 1.0 × 10^16^ s^–1^ for ultraviolet radiation)^68^ with the temperature varying from 130 to 230 K, as shown in Figure 7. The recovery times calculated for Mn@b_64_-COCl_2_ are already approaching 0 s above 150 K, under ultraviolet light, which attests to the use of the sensor at extremely low temperatures. Under yellow light, this occurs around 170 K. However, when we use the tentative frequency corresponding to the infrared range only from 200 K onward, the time is less than 1 s. The results show that the system is suitable for application as a fast response sensor for successive detection of COCl_2_ gas, even under radiation at different wavelengths and even at extreme temperatures.

### UV–Vis Spectroscopy Analysis

**Figure 8:**
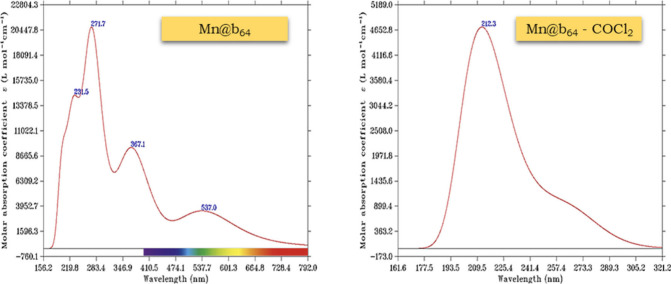
UV–vis spectra of the Mn@b_64_ and Mn@b_64_-COCl_2_ systems.

Several potential applications of pristine and modified BN nanocages have been widely explored in the literature, such as their use in materials for optical sensors.^69,70^ To investigate the possibility of such an application for phosgene detection, UV–vis spectra were estimated by using TD-DFT calculations, with 100 roots and the same level of theory. Figure 8 and Table 3 illustrate the UV–vis absorption spectra and the main electronic transitions of the Mn@b_64_ nanocage (in its most stable spin multiplicity, a sextet) before and after interaction with the phosgene molecule.^44,65^ The pure nanocage presents four absorption peaks: the first appearing at 231.5 nm, with an excitation energy *E* = 5.4 eV and an oscillator strength *f* = 0.08, with a greater contribution of the H(α) → L(α) electronic transition (60%); the second at 271.7 nm, corresponding to an excitation energy *E* = 4.4 eV and an oscillator force *f* = 0.17, which is associated with the transition of electrons from the HOMO orbitals to the LUMO orbitals [H(β) → L(β) (52.4%) and H(α) → L(α) (42%)]; the third at 367.1 nm, with *E* = 3.4 eV and with lower strength (*f* = 0.916), given the greater contribution of the transition of a β electron from the HOMO to the LUMO (74.6%); and the fourth in the visible light region (537 nm), with *E* = 2.3 eV and *f* = 0.08.

**Table 3:** Wavelengths (λ_max_), Oscillator Strengths (*f*), Energies (*E*), and Main Electronic Transitions Associated with the Absorption Peaks of the Mn@b_64_ and Mn@b_64_-COCl_2_ Systems

system	λ_max_ (nm)	*f*	*E* (eV)	transition	
Mn@b_64_	231.5	0.08	5.463	H(α) → L(α)	60.1%
				H(β) → L(β)	21.4%
	271.7	0.17	4.464	H(α) → L(α)	41.0%
				H(β) → L(β)	52.4%
	367.1	0.16	3.435	H(α) → L(α)	22.8%
				H(β) → L(β)	74.6%
	537.0	0.08	2.295	H(α) → L(α)	72.5%
				H(β) → L(β)	26.9%
Mn@b_64_-COCl_2_	210.6	0.011	5.9	H(β) → L(β)	36%
				H – 1(β) → L + 1(β)	26%
				H – 2(β) → L + 2(β)	10%

After phosgene adsorption, the Mn@b_64_-COCl_2_ system presents a maximum absorbance peak (λ_max_ = 210.6 nm), corresponding to an excitation energy *E* = 5.9 eV and an oscillator strength *f* = 0.011, which is associated with the H(β) → L(β) (36%), H – 1(β) → L + 1(β) (26%), and H – 2(β) → L + 2(β) (10%) transitions. We observed that with the adsorption of phosgene, there was a shift in the maximum absorption peak (λ_max_) of the nanocage to regions of higher UV energy and suppression of absorption in the visible region. The data indicate that the Mn@b_64_ nanocage is optically active for applications in the production of COCl_2_ gas optical sensing devices.

### Interference Analysis

To evaluate the influence of the presence of water and other molecules on the adsorption of COCl_2_ in Mn@b_64_ nanocages, the structures of water and some interfering gases commonly used in previous studies reported in the literature^71,72^ (H_2_, CH_4_, CO_2_, NH_3_, N_2_, CO, H_2_S, and N_2_O) were optimized with the same level of theory and then adsorbed on the nanocage surface. Parameters such as the adsorption energy (*E*_ads_), recovery time (τ), energy gap (*E*_gap_), gap variation (Δgap), work function (Φ), work function variation (ΔΦ), sensitivity (as a function of the electrical conductivity (*S_σ_*) and work function (*S_j_*)), and selectivity coefficient for both parameters (*k_σ_* and *k_j_*) were calculated and are listed in Table 4, considering the best values among the α and β orbitals of each system. The results show that the gases N_2_, CO, and CO_2_ adsorb on the nanocage with *E*_ads_ > 0, thereby generating weak and unstable interactions with the cage. The gases H_2_ and CH_4_ have an adsorption energy of less than −0.3 eV, indicating the occurrence of physisorption with van der Waals force-type interactions. These gases also showed low Δgap values (1.87% and 5.03%, respectively). The water and the other gases studied (H_2_O, NH_3_, H_2_S, and NO_2_) were shown to interact through moderate chemical adsorption with Mn@b_64_, thus generating a recovery time that goes from 5 ns to 1.5 min. On the other hand, the electronic sensitivity (Δgap) of the nanocage to phosgene is higher than that of water and all interfering gases. Therefore, the interfering agent with the greatest response is N_2_O gas (30.2%). The Mn@b_64_-COCl_2_ system also presents greater work function variation compared with the interferent molecules studied.

**Table 4:** Adsorption Energies (*E*_ads_), Recovery Times (τ), Energy Gaps (*E*_gap_), Variations of the Gap Energy (Δgap), Work Functions (Φ), Variations of the Work Function (ΔΦ), Sensitivities (*S_σ_* and *S_j_*), and Selectivity Coefficients (*k_σ_* and *k_j_*) for the Adsorption of the Interfering Molecules on Mn@b_64_

Mn@b_64_- gas	*E*_ads_	τ	*E*_gap_ (eV)	Δgap (%)	*S_σ_*	*k_σ_*	Φ (eV)	ΔΦ (%)	*S_j_*	*k_j_*
COCl_2_	–0.48	1.29 μs	5.04 ^β^	69.13	2.54 × 10^17^	1	4.97	24.87	5.36 × 10^16^	1
H_2_	–0.02	21.78 fs	3.26 ^α^	1.87	2.21	1.15 × 10^17^	3.46	13.07	6.12 × 10^8^	8.75 × 10^7^
CH_4_	–0.1	489.58 fs	3.13 ^β^	5.03	1.75 × 10	1.45 × 10^16^	3.86	3.14	1.29 × 10^2^	4.17 × 10^14^
CO_2_	0.13	–	2.63 ^β^	11.74	9.05 × 10^2^	2.81 × 10^14^	4.56	14.45	5.21 × 10^9^	1.03 × 10^7^
NH_3_	–0.94	1.28 min	3.36 ^β^	12.75	1.62 × 10^3^	1.57 × 10^14^	3.68	7.54	1.17 × 10^5^	4.57 × 10^11^
N_2_	0.11	–	3.53 ^β^	18.46	4.44 × 10^4^	5.73 × 10^12^	4.63	16.21	7.93 × 10^10^	6.76 × 10^5^
CO	0.24	–	3.54 ^β^	18.79	5.39 × 10^4^	4.72 × 10^12^	4.58	15.08	1.38 × 10^10^	3.89 × 10^6^
H_2_O	–0.69	4.57 ms	3.56 ^β^	19.46	7.95 × 10^4^	3.20 × 10^12^	3.85	3.27	1.56 × 10^2^	3.43 × 10^14^
H_2_S	–0.34	5.56 ns	3.57 ^β^	19.80	9.66 × 10^4^	2.63 × 10^12^	3.87	2.89	86.8	6.18 × 10^14^
N_2_O	–0.54	13.34 μs	3.88 ^β^	30.20	4.02 × 10^7^	6.32 × 10^9^	4.62	16.08	6.53 × 10^10^	8.21 × 10^5^

The sensitivity (*S*) and the selectivity coefficient (*k*) were calculated for the two parameters individually: electronic sensor (which uses electrical conductivity σ) and work function sensor (which uses current density *j*). Therefore, κ represents the sensitivity ratio between the reaction of COCl_2_ and an interfering gas. In other words, selectivity is the ability of a gas sensor to detect a specific gas in a mixture of gases. With regard to selectivity, we observed that it was possible to differentiate COCl_2_ from water and all interfering gases studied (for both conductometric and work function sensing) and that the highest electronic selectivity coefficient was observed for COCl_2_ with H_2_ (*k_σ_* = 1.15 × 10^17^) and the lowest for CO with N_2_O (*k_σ_* = 6.32 × 10^9^). However, when it comes to work function sensors, the highest and lowest selectivity were recorded for COCl_2_ with H_2_S (*k_j_* = 6.18 × 10^14^) and COCl_2_ with N_2_O (*k_j_* = 8.21 × 10^5^). It is worth mentioning that the higher the selectivity coefficient, the greater the differentiation between gases. Finally, based on the data found, it appears that the Mn@b_64_ nanocage is capable of selectively detecting COCl_2_ gas, in a mixture of others and even in the presence of water molecules, in both electronic and sensor-type applications.

### Stability and Effect of Humidity

To address the effect of humidity on the adsorption process, molecular dynamics (MD) simulations were performed using the xTB method by immersing the Mn@b_64_ nanocage with adsorbed COCl_2_ in a “water box” containing 40 H_2_O molecules. This setup simulates a high-humidity environment to evaluate the stability of the adsorption system. The simulation was conducted for 250 ps with an integration time step of 25 fs, allowing detailed tracking of the system’s behavior over time.

**Figure 9:**
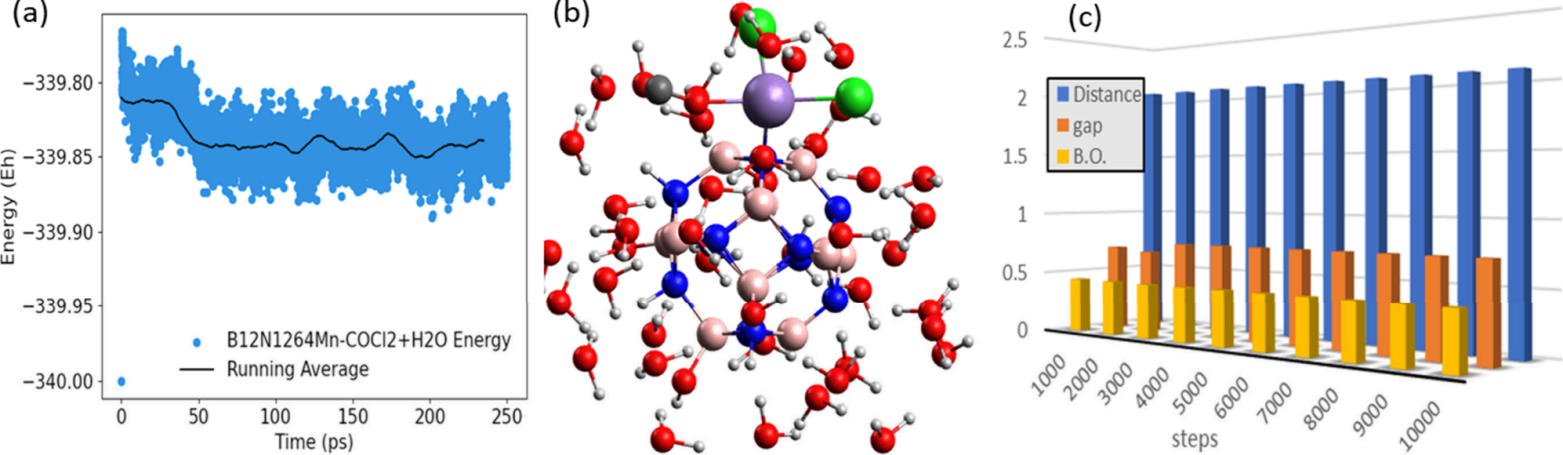
MD trajectory performed (a) in a system with Mn@b_64_-COCl_2_ inserted in a medium with 40 water molecules (b). (c) Graphic showing the energy gap (*E*_gap_), cage/gas, bond distance (Distance), and bond order (B.O.) between the C of the gas and the Mn of the nanocage along the MD.

The results, presented in Figure 9a, reveal minimal energy variations (0.05 hartree) over 10 000 steps, confirming the system’s stability throughout the trajectory. These small fluctuations correspond to adjustments in the relative positioning of COCl_2_ on the nanocage surface with the molecule remaining adsorbed and the nanocage intact during the entire simulation. Figure 9b illustrates the final configuration of the system after 250 ps, demonstrating that the phosgene molecule remains bound despite the surrounding aqueous environment. This approach demonstrates the resistance of the adsorption system to high humidity, highlighting the robust interaction between the cage and COCl_2_ under dynamic conditions, as we used for the analysis of N_2_O in a preview work.^63^ Molecular dynamics is a well-established method for evaluating dynamic stability.^44,47,49^

Figure 9c shows the monitoring of the parameters: energy gap (*E*_gap_), bond distance between the cage and gas (*d*_C–Mn_), and the bond order between the C of the gas and the Mn of the nanocage (B.O.) during the MD. The calculated parameters, *E*_gap_, *d*_O–Mn_, and B.O., did not present significant changes throughout the test. This effectively shows that the adsorption of the gas on the surface of the Mn@b_64_ nanocage does not break down over time and is not affected by humidity. Thus, the material presented here is suitable for application in the development of a device for sensing COCl_2_ gas, being efficient even in environments with high humidity.

Table 5 shows the performance of some materials for COCl_2_ adsorption reported in the literature,^19,28,58^ as well as the results of Mn@b_64_ found here. It is possible to observe that the Mn@b_64_ nanocage presents the highest sensitivity for the gas (Δgap = 69.13%) compared to the other systems, with the exception of the graphene nanosheets presented in the work of Deji et al.^19^ However, the Mn@b_64_ nanocage presented a moderate adsorption energy (*E*_ads_ = 0.48 eV), compared to the nanosheets (*E*_ads_ = −9.82 eV), which allows a shorter recovery time (1.29 μs) and nanocage application in cyclic gas detections. Therefore, it is superior to all of the phosgene detection systems presented.

**Table 5:** Comparison of the Phosgene Detection Performance of Mn@b_64_ and Theoretical Results from the Literature

sensor	functional	*E*_ads_ (eV)	ΔΦ (%)	ref
Sc-BNNT	B3LYP	–17.62	45.6	Beheshtian et al.^22^
P-doped graphene nanoribbons (AGNR)	PBE	–9.82	94.2	Deji et al.^19^
B_12_N_12_	B3LYP	–0.098^a^,^b^	20.61	Hussain at al.^28^
Cu@b_66_		–0.017^a^,^b^	8.30	
Cu@b_64_		–0.176	25.24	
B_12_N_12_	B3LYP	–0.070	18.48	Shakerzadeh et al.^58^
AlB_11_N_12_		–0.940	45.54	
GaB_11_N_12_		–0.561	26.52	
B_16_N_16_		–0.030	10.70	
AlB_15_N_16_		–0.928	49.37	
GaB_15_N_16_		–0.530	30.03	
Al_12_N_12_	M06-2X, B97D, B3LYP	–0.816	13.00	Padash et al.^73^
Al_12_P_12_		–0.272	1.88	
B_12_N_12_		–0.272	11.60	
B_12_P_12_		–0.272	0.36	
Al_12_N_12_	B3LYP, PBE0, ωB97X-D	–0.780	46.00	Louis et al.^27^
Ca_12_O_12_		–4.530	51.20	
Mg_12_O_12_		–1.210	47.90	
Mn@b_64_	B3LYP-D3	–0.480	69.13	this work

a Value calculated without BSSE correction.

b Kilojoules per mole to electronvolts.

The theoretical results obtained in this study are relevant and are on the verge of being experimentally investigated in electrochemical sensors for practical application. It is worth mentioning that electrochemical studies have already been carried out using fullerene nanomaterials^74^ for application in gas sensors. In this sense, the synthesis and electrochemical application of B_12_N_12_ nanocages and derivatives as sensors can experimentally validate the theoretical findings and reveal a promising line of gas sensor development.

## Conclusion

Using DFT, implemented in the ORCA program, a detailed investigation was carried out on the applicability of B_12_N_12_ nanocages, both pure and modified with first-row transition metals (Sc–Zn), in the sensing of the toxic gas phosgene. The nanocages were modified with the metals in five different configurations (TMB_11_N_12_, B_12_N_11_TM, TM@b_64_, TM@b_66_, and TM@B_12_N_12_), allowing us to evaluate both the influence of the nature of the metal and its positioning in the structure on the adsorption of the gas. Geometric, electronic, charge, interaction, and adsorption energies were calculated, revealing that phosgene is adsorbed preferentially by oxygen, interacting with the TM or boron of the nanocages. In certain systems, such as B_12_N_11_Sc, B_12_N_11_Ti, and others, dissociation of the COCl_2_ molecule was observed, forming COCl + Cl or CO + 2Cl.

The interaction energies showed that the doped systems presented stronger cage/gas cohesion, followed by the decorated and encapsulated systems, with the strongest interaction observed for TiB_11_N_12_ (*E*_int_ = −10.16 eV). The electronic analysis indicated a revelation in the HOMO–LUMO gap with an increase in the conductivity and reactivity of the modified nanocages. The quantum descriptors identified the decorated systems Mn@b_64_-COCl_2_ and Fe@b_64_-COCl_2_ as the most stable of the adsorption series and spontaneous for interaction with COCl_2_ (higher hardness, lower electrophilicity, and negative *ΔG*_ads_); on the other hand, they showed that COCl_2_ did not adsorb spontaneously (*ΔG*_ads_ > 0) in the systems with an encapsulated TM (TM@B_12_N_12_), except when encapsulated with Cu and Zn, due to their differentiated properties.

The charge study revealed that the gas/nanocage systems function as Lewis acid–base pairs and the cage transfers charge to the gas. The Δgap revealed that the Mn@b_64_ and Fe@b_64_ systems present higher values of electronic sensitivity to phosgene (124.28% and 69.13%, respectively). However, Mn@b_64_ presented moderate adsorption energy *E*_ads_ = −0.48 eV, lower than that of Fe@b_64_ (*E*_ads_ = −0.99 eV), with a recovery time of 1.29 μs, which prevents its spontaneous desorption and makes it more suitable for cyclic detection and fast response sensors. Mn@b_64_ presented a work function variation of 25% and was optically active in the TD-DFT analysis, with a shift in the maximum absorption peak of the UV spectrum, after the adsorption of phosgene. We compared the performance of some materials for COCl_2_ adsorption found in the literature, and our results were superior to those of all reported phosgene detection systems. Furthermore, Mn@b_64_ was selective to phosgene, capable of detecting it even in the presence of interferents such as H_2_, CH_4_, CO_2_, NH_3_, N_2_, CO, H_2_S, N_2_O, and water. These results highlight the potential of nanocages, especially Mn@b_64_, for application in electrical, optical, and work function sensors for detecting phosgene gas, with high sensitivity and selectivity even in environments with high humidity.

## Supporting Information Available

Transition metal spin multiplicity for the most stable TM-modified nanocages and HOMO energies (*E*_H_), LUMO energies (*E*_L_), and energy gaps (*E*_gap_) calculated for pure and modified B_12_N_12_ nanocages, before and after the adsorption of COCl_2_ gas (PDF)
