# Supplementary material for: Theoretical Study of a Transition Metal-Modified B12N12 Nanocage for COCl2 Detection: Advances toward High-Sensitivity Materials for Phosgene Sensing
Source: Langmuir. 2025 Mar 5;41(11):7396–409. doi: 10.1021/acs.langmuir.4c04850 (PMC11948460; doi:10.1021/acs.langmuir.4c04850)
Supplement: Supplementary file 1 — la4c04850_si_001.pdf [file la4c04850_si_001.pdf]

Supporting Information

Theoretical Study of Transition Metal-Modified $B_{12}N_{12}$ Nanocage for $COCl_2$ Detection: Advances Toward High-Sensitivity Materials to Phosgene Sensing

Natanael de Sousa Sousa,[a*] and Jaldyr de Jesus Gomes Varela Júnior[a]

Affiliations

[a] *Universidade Federal do Maranhão, 65080-805, São Luís, MA, Brazil.*

* Corresponding Author. E-mail address 83.natan@gmail.com

Table of contents

Number of pages: 03

Number of tables: 02

Number of figures: 01

Figure S1 – Transition metal spin multiplicity for the most stable TM-modified nanocages.

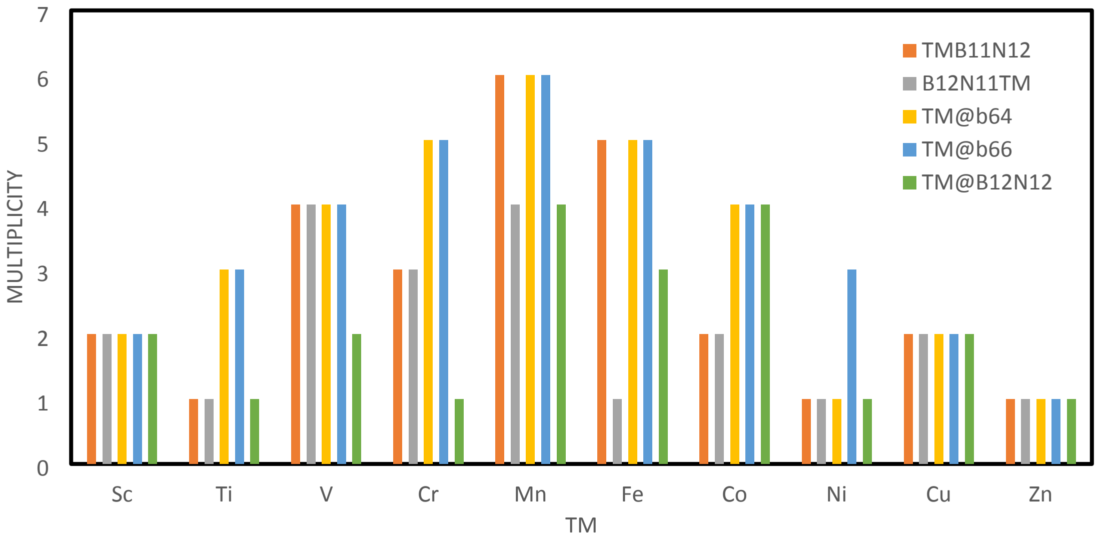

Table S1 – Molecular Orbital (O), HOMO Energy (E_H), LUMO Energy (E_L) and energy gap (E_{gap}) of pure and modified $B_{12}N_{12}$ nanocages.

**Systems**	**O**	**E_H (eV)**	**E_L (eV)**	**E_{gap} (eV)**	**Systems**	**O**	**E_H (eV)**	**E_L (eV)**	**E_{gap} (eV)**
$B_{12}N_{12}$	α	-7.63	-0.75	6.88					
$ScB_{11}N_{12}$	α	-6.75	-2.49	4.26	$FeB_{11}N_{12}$	α	-6.72	-2.25	4.46
$B_{12}N_{11}Sc$	α	-5.08	-2.47	2.61	$B_{12}N_{11}Fe$	β	-5.36	-2.20	3.16
Sc@b_{64}	α	-4.37	-1.88	2.49	Fe@b_{64}	α	-5.06	-2.30	2.76
Sc@b_{66}	β	-4.89	-2.34	2.55	Fe@b_{66}	α	-4.98	-1.74	3.24
Sc@$B_{12}N_{12}$	α	-4.00	-1.81	2.19	Fe@$B_{12}N_{12}$	β	-5.06	-1.06	3.99
$TiB_{11}N_{12}$	α	-5.35	-2.28	3.07	$CoB_{11}N_{12}$	α	-7.06	-3.18	3.88
$B_{12}N_{11}Ti$	β	-5.52	-2.60	2.92	$B_{12}N_{11}Co$	α	-6.23	-2.27	3.96
Ti@b_{64}	α	-4.70	-2.14	2.56	Co@b_{64}	α	-4.95	-1.85	3.09
Ti@b_{66}	β	-5.00	-2.30	2.70	Co@b_{66}	β	-6.23	-2.68	3.56
Ti@$B_{12}N_{12}$	α	-4.66	-1.62	3.04	Co@$B_{12}N_{12}$	α	-4.37	-1.23	3.14
$VB_{11}N_{12}$	β	-7.11	-2.13	4.98	$NiB_{11}N_{12}$	β	-7.32	-3.36	3.96
$B_{12}N_{11}V$	α	-5.53	-2.48	3.04	$B_{12}N_{11}Ni$	α	-4.92	-2.39	2.53
V@b_{64}	β	-5.65	-2.12	3.54	Ni@b_{64}	α	-5.72	-2.54	3.18
V@b_{66}	β	-5.00	-2.26	2.74	Ni@b_{66}	β	-6.44	-2.65	3.80
V@$B_{12}N_{12}$	α	-4.69	-1.31	3.38	Ni@$B_{12}N_{12}$	α	-5.84	-0.97	4.87
$CrB_{11}N_{12}$	β	-7.14	-2.20	4.95	$CuB_{11}N_{12}$	α	-6.89	-4.84	2.05
$B_{12}N_{11}Cr$	β	-5.56	-2.45	3.12	$B_{12}N_{11}Cu$	α	-5.16	-2.40	2.76
Cr@b_{64}	α	-5.65	-2.38	3.27	Cu@b_{64}	β	-6.75	-2.57	4.18
Cr@b_{66}	β	-4.86	-2.17	2.69	Cu@b_{66}	β	-6.75	-2.55	4.20
Cr@$B_{12}N_{12}$	α	-4.98	-1.46	3.53	Cu@$B_{12}N_{12}$	α	-4.08	-1.37	2.71
$MnB_{11}N_{12}$	β	-7.18	-2.66	4.53	$ZnB_{11}N_{12}$	α	-7.09	-2.39	4.70
$B_{12}N_{11}Mn$	β	-4.70	-1.94	2.76	$B_{12}N_{11}Zn$	β	-5.60	-2.66	2.94
Mn@b_{64}	β	-5.47	-2.49	2.98	Zn@b_{64}	α	-6.37	-1.85	4.52
Mn@b_{66}	α	-5.06	-1.82	3.24	Zn@b_{66}	α	-6.37	-1.85	4.52
Mn@$B_{12}N_{12}$	α	-5.34	-1.12	4.22	Zn@$B_{12}N_{12}$	α	-3.81	-1.64	2.17

Table S2 – HOMO (E_H), LUMO (E_L) and energy gap (E_{gap}) of the adsorption of $COCl_2$ gas in bare and TM-modified $B_{12}N_{12}$ nanocages.

Systems	E_H (eV)	E_L (eV)	E_{gap} (eV)	Systems	E_H (eV)	E_L (eV)	E_{gap} (eV)
$B_{12}N_{12}$-$COCl_2$	-7.41	-2.03	5.38				
$ScB_{11}N_{12}$-$COCl_2$	-6.41	-3.88	2.53	$FeB_{11}N_{12}$-$COCl_2$	-6.27	-3.63	2.64
$B_{12}N_{11}Sc$-$COCl_2$	-5.80	-3.42	2.39	$B_{12}N_{11}Fe$-$COCl_2$	-5.09	-2.79	2.30
Sc@b_{64}-$COCl_2$	-5.37	-2.62	2.75	Fe@b_{64}-$COCl_2$	-7.60	-1.41	6.19
Sc@b_{66}-$COCl_2$	-6.20	-2.72	3.48	Fe@b_{66}-$COCl_2$	-7.40	-2.62	4.78
Sc@$B_{12}N_{12}$-$COCl_2$	-3.75	-2.80	0.95	Fe@$B_{12}N_{12}$-$COCl_2$	-4.81	-2.27	2.53
$TiB_{11}N_{12}$-$COCl_2$	-5.59	-2.96	2.63	$CoB_{11}N_{12}$-$COCl_2$	-6.55	-3.34	3.21
$B_{12}N_{11}Ti$-$COCl_2$	-5.78	-2.55	3.23	$B_{12}N_{11}Co$-$COCl_2$	-6.07	-3.17	2.89
Ti@b_{64}-$COCl_2$	-6.23	-2.93	3.30	Co@b_{64}-$COCl_2$	-7.23	-2.42	4.81
Ti@b_{66}-$COCl_2$	-5.78	-2.32	3.46	Co@b_{66}-$COCl_2$	-6.77	-2.27	4.50
Ti@$B_{12}N_{12}$-$COCl_2$	-4.36	-2.55	1.81	Co@$B_{12}N_{12}$-$COCl_2$	-4.05	-2.74	1.31
$VB_{11}N_{12}$-$COCl_2$	-7.44	-5.40	2.04	$NiB_{11}N_{12}$-$COCl_2$	-6.79	-3.16	3.63
$B_{12}N_{11}V$-$COCl_2$	-6.25	-2.64	3.61	$B_{12}N_{11}Ni$-$COCl_2$	-4.52	-2.40	2.12
V@b_{64}-$COCl_2$	-6.15	-2.32	3.83	Ni@b_{64}-$COCl_2$	-5.79	-2.79	2.99
V@b_{66}-$COCl_2$	-5.58	-2.19	3.39	Ni@b_{66}-$COCl_2$	-7.37	-2.79	4.58
V@$B_{12}N_{12}$-$COCl_2$	-4.49	-2.27	2.22	Ni@$B_{12}N_{12}$-$COCl_2$	-5.48	-2.35	3.13
$CrB_{11}N_{12}$-$COCl_2$	-6.77	-3.45	3.32	$CuB_{11}N_{12}$-$COCl_2$	-6.46	-4.14	2.32
$B_{12}N_{11}Cr$-$COCl_2$	-5.28	-3.16	2.12	$B_{12}N_{11}Cu$-$COCl_2$	-4.85	-2.38	2.46
Cr@b_{64}-$COCl_2$	-5.29	-2.92	2.36	Cu@b_{64}-$COCl_2$	-7.01	-2.05	4.95
Cr@b_{66}-$COCl_2$	-4.79	-2.73	2.06	Cu@b_{66}-$COCl_2$	-7.11	-2.01	5.10
Cr@$B_{12}N_{12}$-$COCl_2$	-4.69	-2.40	2.30	Cu@$B_{12}N_{12}$-$COCl_2$	-5.17	-1.60	3.57
$MnB_{11}N_{12}$-$COCl_2$	-6.52	-4.46	2.06	$ZnB_{11}N_{12}$-$COCl_2$	-6.93	-2.15	4.78
$B_{12}N_{11}Mn$-$COCl_2$	-6.35	-4.62	1.73	$B_{12}N_{11}Zn$-$COCl_2$	-5.16	-3.02	2.14
Mn@b_{64}-$COCl_2$	-7.49	-2.45	5.04	Zn@b_{64}-$COCl_2$	-6.14	-1.99	4.15
Mn@b_{66}-$COCl_2$	-7.36	-2.54	4.81	Zn@b_{66}-$COCl_2$	-6.16	-2.03	4.13
Mn@$B_{12}N_{12}$-$COCl_2$	-4.51	-2.36	2.15	Zn@$B_{12}N_{12}$-$COCl_2$	-6.03	-2.	3.15
